# Diurnal Temperature Range in Relation to Daily Mortality and Years of Life Lost in Wuhan, China

**DOI:** 10.3390/ijerph14080891

**Published:** 2017-08-08

**Authors:** Yunquan Zhang, Chuanhua Yu, Jin Yang, Lan Zhang, Fangfang Cui

**Affiliations:** 1 Department of Preventive Medicine, School of Health Sciences, Wuhan University, 185 Donghu Road, Wuhan 430071, China; Yun-quanZhang@whu.edu.cn (Y.Z.); jinan0218@163.com (J.Y.); cuifangfang@whu.edu.cn (F.C.); 2 Global Health Institute, Wuhan University, 8 Donghunan Road, Wuhan 430072, China; 3 Office of Chronic Disease, Hubei Provincial Center for Disease Control and Prevention, 6 Zhuodaoquan Road, Wuhan 430079, China; hbcdczl@163.com

**Keywords:** climate change, diurnal temperature range, mortality, years of life lost, China

## Abstract

Diurnal temperature range (DTR) is an important meteorological indicator associated with global climate change, and has been linked with mortality and morbidity in previous studies. To date, however, little evidence has been available regarding the association of DTR with years of life lost (YLL). This study aimed to evaluate the DTR-related burden on both YLL and mortality. We collected individual records of all registered deaths and daily meteorological data in Wuhan, central China, between 2009 and 2012. For the whole population, every 1 °C increase in DTR at a lag of 0–1 days was associated with an increase of 0.65% (95% CI: 0.08–1.23) and 1.42 years (−0.88–3.72) for mortality and YLL due to non-accidental deaths, respectively. Relatively stronger DTR-mortality/YLL associations were found for cardiovascular deaths. Subgroup analyses (stratified by gender, age, and education level) showed that females, the elderly (75+ years old), and those with higher education attainment (7+ years) suffered more significantly from both increased YLL and mortality due to large DTR. Our study added additional evidence that short-term exposure to large DTR was associated with increased burden of premature death using both mortality incidence and YLL.

## 1. Introduction

During the last few decades, the association of daily mortality with ambient temperature has been extensively investigated throughout the world, using time-series or case-crossover designs [1,2,3,4]. According to a recent multi-country study [5], non-optimum temperatures (i.e., cold and heat) contributed to 7.71% (95% CI: 7.43–7.91) of daily total mortality incidence between 1985 and 2012. Also, there is increasing evidence that an aging population could substantially enhance the heat-related mortality burden [6,7]. In the context of global climate change and population aging, burden assessment from temperature extremes and other unstable weather events would be of great significance from the public health perspective [8].

As another important meteorological indicator associated with global climate change [9], diurnal temperature range (DTR) is defined as the difference between daily maximum and minimum temperature, which describes the within-day temperature variability and reflects weather stability [10,11,12]. During the past decade, DTR-mortality association assessment has received increasing interest in environmental epidemiology, linking large DTR with increased mortality risk in geographical locations with different climate characteristics [13,14,15,16]. However, DTR-related evidence focusing on vulnerable subgroups and seasonal patterns, as well as cause-specific differences, is still relatively limited compared with available findings from cold- and heat-related studies [17].

As an alternative estimate for mortality incidence in quantifying premature deaths [18], years of life lost (YLL) is an emerging health endpoint when assessing the burden of ambient temperature [19,20]. Furthermore, both of these studies, and several air pollution-YLL investigations, have recommended YLL as a supplementary assessment for mortality because of more informative and accurate estimate through taking both death counts and the differences in death ages into account [21,22,23]. Additionally, estimation of YLL combined with the number of deaths could contribute a lot to outlining a more comprehensive understanding when evaluating the global, regional, and national mortality burden due to premature deaths [24,25]. To date, little epidemiologic evidence has been available worldwide regarding the relation between DTR and YLL, which may have limited more comprehensive understanding in the health burden associated with DTR, thus hampering the decision-making process of public health authority in coping with global climate change. Hence, we performed this time series study in Wuhan, central China, and aimed to examine the burden of DTR on both mortality and YLL. Subgroup analyses were also conducted stratified by individual characteristics (e.g., gender and age), so as to identify the vulnerable subjects to ambient DTR.

## 2. Materials and Methods

### 2.1. Study Area and Population

This study was conducted in the capital city of Hubei Province, central China—Wuhan. Known as the largest city in central China, Wuhan is located in the middle of the Yangzi River and has a typical subtropical monsoon climate [26,27]. Two urban districts, namely Jiang’an and Qiaokou, were selected as the study area, with a total of about 1.22 million permanent residents in 2010 and an urban area of 111.7 km^2^.

### 2.2. Data Collection

#### 2.2.1. Mortality and YLL Data

Daily mortality data from 1 January 2009 to 31 December 2012 were obtained from the Hubei Provincial Center for Disease Control and Prevention. Causes of death were coded in accordance with ICD-10 (the Tenth Revision of the International Classification of Diseases). Daily non-accidental death cases (ICD-10: A00-R99) were included in our databases, and were divided into several mortality categories [28]: cardiorespiratory (I00–I99 and J00–J99), cardiovascular (I00–I99), respiratory (J00–J99), stroke (I60–I69), and ischemic heart disease (IHD: I20–I25). Non-accidental mortality data were further classified into several subgroups stratified by gender, age (0–74 years, 75+ years), and education level (EL: 0–6 years, 7+ years).

YLL calculation in the present study was based on the Chinese national life table for the year 2012 (Supplementary Material Table S1), which was obtained from Global Health Observatory (GHO) data on the WHO website (http://www.who.int/gho/countries/chn/en/). As illustrated in previous studies [21,29], we first obtained YLL for each death by matching age and sex to the reference life table, and summed the YLL of all death cases on each day into a time series of daily YLLs. Corresponding to mortality data, we also calculated daily YLLs for various mortality categories and subgroups.

#### 2.2.2. Ambient Meteorological Data

Daily meteorological data between 2009 and 2012, including maximum, mean, and minimum temperature (°C), DTR(°C), mean relative humidity (%), mean wind speed (m/s), sunshine duration (h), and mean atmospheric pressure (hPa), were collected from the China Meteorological Data Network (http://data.cma.cn), which is run by the China Meteorological Administration and provides a publicly available data sharing service.

### 2.3. Data Analysis

A standard time-series generalized linear model (GLM) [30] was used to conduct the data analysis. We applied a quasi-Poisson regression allowing for overdispersed daily death counts when assessing DTR-mortality relations and specified a Gaussian process for YLL, since daily YLLs generally followed a normal distribution [21,23]. Our preliminary analyses identified approximately linear exposure-response associations of ambient DTR with mortality and YLL (Supplementary Material Figure S1), which was in accordance with previous epidemiologic evidence [10,31,32]. Therefore, a linear term for DTR was included in our final GLM regression analysis.

According to previous studies, a natural cubic spline with 7 degrees of freedom (*df*) per year for calendar time was used to adjust for the long-term trends and seasonality. A flexible “cross-basis” function for daily mean temperature (MeanT) created by distributed lag non-linear model (DLNM) [33,34] was incorporated into the GLM regression to control for the lagged and nonlinear effects of ambient temperatures. Specifically, MeanT was defined by a combination of a natural cubic spline with 3 internal knots at equally spaced percentiles (25th, 50th, and 75th) for temperature space and a natural cubic spline with 2 internal knots at equally spaced log-values (approximately 1.4 and 5.5 days) for lag space. We chose 21 days as the maximum lag to completely capture the overall temperature effect and adjust for any potential harvesting. These modeling choices were motivated by a previous multi-country time-series study [35].

To further eliminate the confounding effects of other meteorological variables, the current day’s mean relative humidity (MeanRh), sunshine duration (Sunshine), mean wind speed (WS), and atmospheric pressure (AP) were also introduced in the GLM as covariates using a natural cubic spline with 3 *df* [12]. Additionally, day of the week (DOW) and public holiday (PH) were also controlled as indicator variables [36,37]. Hence, the core models are given as following:

$$\begin{array}{ll} \mathrm{Log}\left[E\left({\mathrm{death}}_{i}\right)\right] & =\;\alpha \;+\;\beta \mathrm{DTR}\;+\;NS\left(Time_{i},df\;=\;4\;\times \;7\right)\;+\;\gamma {\mathrm{MeanT}}_{t,l}\;\\ & +\;NS\left(MeanRh_{i},df\;=\;3\right)\;+\;\;NS\left(Sunshine_{i},df\;=\;3\right)\;\\ & +\;\;NS\left(WS_{i},df\;\;=\;\;3\right)\;\;+\;NS\left(AP_{i},df\;=\;3\right)\;+\;\delta DOW_{i}\;+\;\varepsilon PH_{i} \end{array}$$

and

$$\begin{array}{ll} E\left({\mathrm{YLL}}_{i}\right)\;=\;\alpha \;+ & \beta \mathrm{DTR}\;+\;NS\left(Time_{i},df\;=\;4\times 7\right)\;+\;\gamma {\mathrm{MeanT}}_{t,l}\;+\;\;NS\left(MeanRh_{i},df\;=\;3\right)\;\\ & +\;\;NS\left(Sunshine_{i},df\;=\;3\right)\;+\;NS\left(WS_{i},df\;=\;3\right)\;+\;NS\left(AP_{i},df\;=\;3\right)\;\\ & +\;\delta DOW_{i}\;+\;\varepsilon PH_{i} \end{array}$$

where *i* is the day of observation (*i* = 1, 2, 3…1461), and death*_i_* and YLL*_i_* are the observed daily death counts and years of life lost, respectively; α is the intercept, and β is the regression coefficient for DTR; MeanT*_t,l_* is the cross-basis matrix of mean temperature (*t*) and lag pattern (*l*) produced by DLNM, γ is the vector of coefficients for MeanT*_t,l_*; *NS* is the natural cubic spline function; δ and ε are the regression coefficients for DOW and PH, respectively.

All the analyses were performed using R software version 3.3.2 (R Development Core Team 2016, Vienna, Austria, http://www.R-project.org), with the “dlnm” package to create the DLNM for mean temperature. The results were expressed as changes (estimates and 95% confidence intervals, CI) in daily mortality (%) and YLL (years) associated with a 1 °C increase in DTR. Two-sided statistical tests were conducted, and effects of *p* < 0.05 were considered statistically significant. To check the robustness of the results, some sensitivity analyses were performed by changing *df* (4–6 per year) in the smoothness of time and *df* (4–6) of natural cubic spline for MeanRh, Sunshine, WS, and AP (Supplementary Materials Tables S2 and S3).

## 3. Results

### 3.1. Data Description

Table 1 describes the distribution characteristics of daily meteorological variables in Wuhan, China, between 2009 and 2012. The average daily mean temperature and relative humidity were 16.8 °C (range: −2.9–35.3 °C), and 76.6% (range: 24–100%), respectively. During the study period, daily DTR varied greatly from 0.9 °C to 19.5 °C, with the mean value of 8.4 °C.

Table 2 summarizes the descriptive statistics of cause-specific and subgroup-specific daily death counts and years of life lost. Daily mean cases from non-accidental, cardiovascular, and respiratory deaths were 21.4, 9.7, and 2.1, respectively, correspondingly contributing to 317.8, 110.4, and 19.7 person years in daily YLL. We observed more deaths and higher YLL among males than females, low-educated persons than those with higher education level. Compared with the elderly (aged over 75 years), the younger group suffered relatively fewer death cases, but much greater YLL. For non-accidental mortality, annual mean deaths changed little between 2009 and 2012, while annual mean YLL decreased gradually from 330.1 in 2009 to 305.4 in 2012 (Supplementary Material Table S4). As illustrated in Supplementary Materials Figure S2, both death cases and YLL peaked in cold months (e.g., December and January) and reached their trough in hot months (e.g., May to August).

### 3.2. Lag Patterns of DTR-Associated Effects

**Figure 1:**
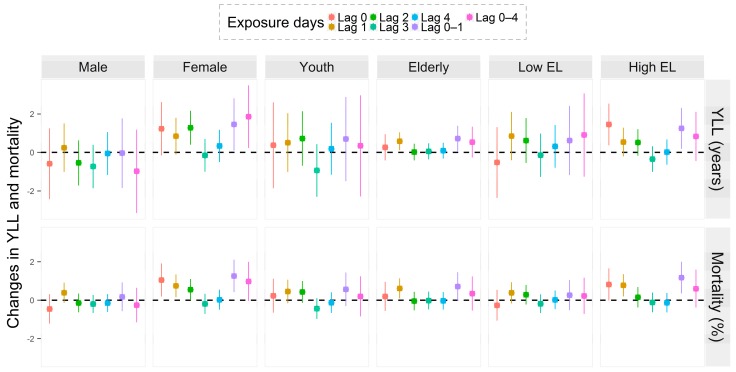
The estimated effects of DTR on subgroup-specific mortality and YLL at different exposure days. The results were presented as changes in daily mortality (%) and YLL (years) associated with a 1 °C increase in DTR. Youth: 0–74 years old; Elderly: 75+ years old; Low EL: 0–6 years’ education level; High EL: 7+ years’ education level.

Figure 1 and Supplementary Materials Figure S3 show the lag patterns of mortality and YLL effects associated with per 1 °C increase in DTR, stratified by different mortality categories, gender, age, and education level. Generally, few effects of DTR on mortality and YLL were observed after days of lag 1. Hence, we used exposure days of lag 0–1 to subsequently estimate the burden of mortality and YLL due to daily changes in DTR.

### 3.3. DTR Effects on Cause-Specific Mortality and YLL

Table 3 calculates the estimated effects of a 1 °C increase in DTR at lag 0–1 days on cause-specific mortality and YLL. For the whole population, higher DTR significantly increased daily non-accidental mortality, especially cardiovascular mortality, with corresponding increases of 0.65% (95% CI: 0.08–1.23) and 1.12% (0.28–1.97) associated with 1 °C increase in DTR. Compared with daily mortality, YLL showed less significant increases in association with DTR.

### 3.4. Subgroup Analysis Stratified by Gender, Age, and Education Level

Table 4 demonstrates the estimated DTR effects on mortality and YLL due to non-accidental and cardiovascular deaths, stratified by subgroups (i.e., gender, age, and education level). Compared with males and younger persons, females and the elderly suffered more significantly and substantially from both increased mortality and YLL in relation to high DTR. The low-educated group, rather than those with higher education attainment, was more strongly affected by the DTR-associated burden on mortality and YLL.

## 4. Discussion

In this ecological study, we examined the burden of both YLL and mortality in relation to short-term DTR exposure in urban China. High DTR was identified as an independent risk factor for YLL and mortality, with some vulnerable subgroups in DTR-associated effects. Our findings could have some important implications for better decision-making and health resource allocation in coping with global climate change in China.

Consistent with most previous evidence [17], our study identified the adverse effects of high DTR on short-term mortality in a humid subtropical city of central China. Using data from the National Morbidity Mortality Air Pollution Study (NMMAPS), a nationwide investigation covering 95 large US communities linked an increase of 0.27% (95% CI: 0.24–0.30%) in non-accidental mortality with a 1 °C increase in DTR [14]. Additionally, another two multi-city time series studies reported the pooled DTR effects of 0.5% (0.3–0.7%) in six Korean cities [38] and 0.18% (0.09–0.28%) in 8 large Chinese cities [32]. In the present study, we observed relatively greater mortality effects associated with DTR, but still much lower than the estimated magnitude in Shanghai between 2001 and 2004 [31]. The regional differences in DTR-associated health effects have been comprehensively demonstrated using multinational data between 1979 and 2010 from 30 cities in East Asia [16], and could be related to several factors, such as climate conditions, population vulnerability, and socioeconomic status of the study population [14,17,38].

As the top leading causes of death in China [39], cardiovascular disease has been previously identified as a mortality category susceptible to several environmental risk factors (e.g., air pollution and temperature extremes). Also, a number of DTR-morbidity/mortality studies found greater effects on cardiovascular deaths [10,31], since greater DTR could cause the increase in heart rate, blood pressure, and oxygen uptake, and further aggravate the workload and burden of the previously harmed heart. Using both YLL and mortality as quantitative estimates for premature deaths, our seasonal and subgroup analyses strengthened the great cardiovascular burden associated with large DTR (Table 3 and Table 4). Additionally, this study revealed strong DTR effects on IHD mortality and YLL, which was in accordance with the previous evidence that DTR was an independent risk factor for coronary heart disease death [40]. For stroke and respiratory mortality/morbidity, while many investigations demonstrated adverse DTR-related impacts [17], insignificant associations were reported in the present study, as well as two prior studies for stroke hospital admission in Northern Portugal [41] and respiratory mortality in Guangzhou, China [10]. Given the potential ecological bias induced by the study design and exposure assignment, more well-designed investigations should be conducted to better understand the differences of health effects between cause-specific outcomes in relation to DTR. Furthermore, to more comprehensively evaluate DTR-related burden on premature death, more research interests should be focused on using YLL as the outcome measurement, in addition to mortality.

Large bodies of previous investigations have differentiated the potential vulnerability differences from subgroups stratified by individual characteristics when assessing temperature-related mortality impact [42,43,44,45,46]. In accordance with most epidemiologic findings, our study demonstrated more significant associations of DTR with mortality and YLL among females and the elderly. Between-gender heterogeneity could be possibly induced by the great differences in biological heredity and social roles [32,38], while the high susceptibility of elder persons could be largely attributable to the continuing deterioration of physical function, sharp decrease in organism immunity, and some certain pre-existing chronic diseases [3,28]. Given the rapid aging process of human population, worldwide cooperation in taking some preventive measures would be of great significance and urgency, so as to minimize the health burden due to inevitable climate change. Compared with gender and age, the potential effect modification of education level (an important indicator of one’s overall socioeconomic status) in DTR-health associations has been less frequently examined by previous studies [17]. Our study found that those with higher education attainment suffered greater burden of morality and YLL, which was contrary to the finding of a prior time-series study in six Korean cities [38]. Furthermore, no significant EL modification between DTR-mortality association was identified in another recent Chinese investigation in Yuxi City, with risk ratio at lag 0–2 of 0.96 (0.88–1.03) for highly-educated persons relative to those with low EL [47]. Since the available evidence is relatively sparse and lacks consistency, large-scale investigations are needed worldwide in more diverse climate zones to further clarify the inconclusiveness in the future.

The present study had some limitations. Firstly, our assessment of temperature exposure across a relatively large urban area was based on only one fixed monitoring site, and failed to account for the intra-urban variation (e.g., spatial distribution), which could potentially introduce some inevitable measurement bias and limit model predictability in the actual effects of temperature on mortality [48]. Future evidence should focus on more tenuous exposure-response patterns using spatiotemporal models. Secondly, we did not adjust for the effects of air pollutants in our main analyses due to data unavailability. However, the results of our study would not be substantially affected, since very close effect estimates were obtained in previous DTR/temperature-mortality studies with and without controlling for air pollutants [14,49,50]. Additionally, the findings of this single-city study should be cautious of directly generalizing to other locations because of the great differences in climate conditions, population vulnerability, and life expectancy.

## 5. Conclusions

In summary, our study added additional evidence that short-term exposure to large DTR was associated with increased burden of premature death using both mortality incidence and years of life lost. Females, the elderly, and highly-educated persons were found to suffer greater DTR-related burden of mortality and YLL. These findings would contribute to public health policy-making and resources allocation in China, so as to reduce the health burden associated with unstable weather patterns, especially for those susceptible subpopulations.

## Figures and Tables

**Table 1:** Distribution characteristics of daily meteorological variables in Wuhan, China, 2009–2012.

Meteorological Variables	Mean ± SD	Range	Percentile				
			P_1_	P_25_	P_50_	P_75_	P_99_
Mean temperature (°C)	16.8 ± 9.6	−2.9–35.3	−0.6	8.2	18.1	25.0	32.8
Minimum temperature (°C)	13.2 ± 9.8	−7.8–31.4	−4.7	4.8	14.1	21.7	29.6
Maximum temperature (°C)	21.6 ± 9.7	0.7–39.1	2.2	13.2	23.1	29.8	37.3
DTR (°C)	8.4 ± 3.9	0.9–19.5	1.5	5.4	8.3	11.0	16.9
Mean relative humidity (%)	76.6 ± 12.4	24–100	39	70	78	86	97
Mean wind speed (m/s)	1.9 ± 1.0	0.2–6.8	0.6	1.2	1.7	2.3	5.2
Sunshine duration (h)	4.5 ± 4.1	0.0–13.2	0.0	0.0	4.5	8.3	11.9
Atmospheric pressure (hPa)	1012.7 ± 9.5	992.7–1037.6	996.7	1004.6	1013.0	1019.7	1033.2

**Table 2:** Descriptive statistics of daily mortality and YLL due to non-accidental deaths, stratified by causes, gender, age, and education level, in Wuhan, China, 2009–2012.

Variables	Mean ± SD	Minimum	P_25_	P_50_	P_75_	Maximum
**Death Counts**						
Non-accidental	21.4 ± 5.9	9	17	21	25	47
Cardiorespiratory	11.8 ± 4.5	1	9	11	15	36
Cardiovascular	9.7 ± 3.8	1	7	9	12	29
Respiratory	2.1 ± 1.6	0	1	2	3	9
Stroke	5.4 ± 2.6	0	4	5	7	14
IHD	3.3 ± 2.0	0	2	3	4	13
Gender						
Male	11.9 ± 3.9	2	9	12	14	29
Female	9.5 ± 3.5	1	7	9	12	24
Age (years)						
0–74	9.3 ± 3.3	0	7	9	11	22
75+	12.0 ± 4.3	2	9	12	15	29
Education level (years)						
0–6	10.7 ± 3.6	2	8	11	13	29
7+	10.2 ± 3.9	2	7	10	13	27
**YLL (years)**						
Non-accidental	317.8 ± 97.2	97.9	246.8	308.3	378.9	730.8
Cardiorespiratory	130.1 ± 54.6	9.9	90.6	123.6	163.8	356.8
Cardiovascular	110.4 ± 48.4	5.4	74.8	105.5	140.8	298.1
Respiratory	19.7 ± 19.4	0	6.6	14.5	27.9	131.7
Stroke	61.4 ± 33.7	0	36.9	57.4	81.4	216.3
IHD	36.6 ± 25.8	0	16.6	32.4	51.7	155.5
Gender						
Male	189.4 ± 73.6	30.4	135.3	179.1	235	489.8
Female	128.4 ± 55.7	9.9	87.6	121.8	160.2	403.7
Age (years)						
0–74	229.4 ± 88.5	0	165.1	218.4	286.1	648.2
75 +	88.4 ± 31.3	8.6	65	86	107.5	205
Education level (years)						
0–6	197.1 ± 73.4	21.6	145.2	189.1	241.5	498.8
7+	104.6 ± 45.2	15.2	71.7	98.6	131.7	331.7

Note: IHD indicates ischemic heart disease.

**Table 3:** Associations of 1 °C increase in DTR at lag 0–1 days with cause-specific mortality and YLL in Wuhan, China during 2009–2012.

Cause	Mortality (%)		YLL (Years)	
	Estimate (95% CI)	*p*-Value	Estimate (95% CI)	*p*-Value
Non-accidental	**0.65 (0.08, 1.23)**	**0.026**	1.42 (−0.88, 3.72)	0.226
Cardiorespiratory	0.73 (−0.03, 1.50)	0.061	0.65 (−0.56, 1.87)	0.294
Cardiovascular	**1.12 (0.28, 1.97)**	**0.009**	0.92 (−0.19, 2.02)	0.104
Respiratory	−1.01 (−2.73, 0.73)	0.253	−0.27 (−0.74, 0.21)	0.272
Stroke	0.84 (−0.27, 1.97)	0.139	0.14 (−0.65, 0.94)	0.724
IHD	1.35 (−0.07, 2.79)	0.062	0.47 (−0.16, 1.11)	0.145

Note: The bold results are statistically significant (*p* < 0.05).

**Table 4:** Subgroup-specific effects of DTR at lag 0–1 days on mortality and YLL due to non-accidental and cardiovascular deaths in Wuhan, China during 2009–2012.

Cause/Subgroups	Mortality (%)		YLL (Years)	
	Estimate (95% CI)	*p*-Value	Estimate (95% CI)	*p*-Value
Non-accidental deaths				
Male	0.17 (−0.58, 0.93)	0.651	−0.04 (−1.84, 1.77)	0.969
Female	**1.26 (0.42, 2.11)**	**0.003**	**1.46 (0.10, 2.82)**	**0.036**
Youth	0.57 (−0.30, 1.44)	0.201	0.70 (−1.49, 2.88)	0.531
Elderly	0.71 (−0.03, 1.46)	0.059	**0.72 (0.05, 1.39)**	**0.034**
Low EL	0.26 (−0.52, 1.05)	0.513	0.62 (−1.18, 2.42)	0.502
High EL	**1.18 (0.36, 2.00)**	**0.005**	**1.25 (0.19, 2.32)**	**0.021**
Cardiovascular deaths				
Male	0.53 (−0.65, 1.71)	0.38	0.02 (−0.88, 0.92)	0.963
Female	**1.74 (0.56, 2.92)**	**0.004**	**0.90 (0.24, 1.56)**	**0.008**
Youth	0.89 (−0.50, 2.30)	0.212	0.31 (−0.68, 1.30)	0.536
Elderly	**1.22 (0.20, 2.26)**	**0.019**	**0.60 (0.14, 1.07)**	**0.011**
Low EL	1.07 (−0.17, 2.32)	0.091	0.29 (−0.61, 1.20)	0.525
High EL	**1.27 (0.14, 2.41)**	**0.027**	0.56 (−0.04, 1.16)	0.069

Note: The bold results are statistically significant (*p* < 0.05). Youth: 0–74 years old; Elderly: 75+ years old; Low EL: 0–6 years’ education level; High EL: 7+ years’ education level.

## Supplementary Materials

The following are available online at www.mdpi.com/1660-4601/14/8/891/s1, Table S1: Age-specific life expectancy for Chinese females and males for the year 2012. Figure S1: Dose-response relationships of DTR at lag 0–1 days (smoothing by a natural cubic spline with *df* = 3) with daily mortality and YLL due to non-accidental and cardiorespiratory deaths in Wuhan, China, 2009-2012. Figure S2: Boxplots for monthly death number and YLL due to non-accidental mortality in Wuhan, China, 2009–2012. Figure S3: The estimated effects of DTR on cause-specific mortality and YLL at different exposure days. Table S2: Sensitivity analyses for DTR-associated effects on YLL and mortality among females, by changing *df* (4–6 per year) in the smoothness of calendar time and *df* (4–6) of natural cubic spline for mean humidity, mean wind speed, sunshine duration, and atmospheric pressure. Table S3: Sensitivity analyses for DTR-associated effects on YLL and mortality among the elderly (75+ years old), by changing *df* (4–6 per year) in the smoothness of calendar time and *df* (4–6) of natural cubic spline for mean humidity, mean wind speed, sunshine duration, and atmospheric pressure. Table S4: Yearly summary distributions of daily death number and YLL during 2009–2012.

## References

[1] Song X., Wang S., Hu Y., Yue M., Zhang T., Liu Y., Tian J., Shang K. (2017). Impact of ambient temperature on morbidity and mortality: An overview of reviews. Sci. Total Environ..

[2] Amegah A.K., Rezza G., Jaakkola J.J. (2016). Temperature-related morbidity and mortality in sub-saharan Africa: A systematic review of the empirical evidence. Environ. Int..

[3] Yu W., Mengersen K., Wang X., Ye X., Guo Y., Pan X., Tong S. (2012). Daily average temperature and mortality among the elderly: A meta-analysis and systematic review of epidemiological evidence. Int. J. Biometeorol..

[4] Bunker A., Wildenhain J., Vandenbergh A., Henschke N., Rocklov J., Hajat S., Sauerborn R. (2016). Effects of air temperature on climate-sensitive mortality and morbidity outcomes in the elderly: A systematic review and meta-analysis of epidemiological evidence. EBioMedicine.

[5] Gasparrini A., Guo Y., Hashizume M., Lavigne E., Zanobetti A., Schwartz J., Tobias A., Tong S., Rocklov J., Forsberg B. (2015). Mortality risk attributable to high and low ambient temperature: A multicountry observational study. Lancet.

[6] Li T., Horton R.M., Bader D.A., Zhou M., Liang X., Ban J., Sun Q., Kinney P.L. (2016). Aging will amplify the heat-related mortality risk under a changing climate: Projection for the elderly in Beijing, China. Sci. Rep..

[7] Chen K., Zhou L., Chen X., Ma Z., Liu Y., Huang L., Bi J., Kinney P.L. (2016). Urbanization level and vulnerability to heat-related mortality in Jiangsu province, China. Environ. Health Perspect..

[8] Basu R., Ostro B.D. (2008). A multicounty analysis identifying the populations vulnerable to mortality associated with high ambient temperature in California. Am. J. Epidemiol..

[9] Makowski K., Wild M., Ohmura A. (2008). Diurnal temperature range over Europe between 1950 and 2005. Atmos. Chem. Phys. Discuss..

[10] Yang J., Liu H.Z., Ou C.Q., Lin G.Z., Zhou Q., Shen G.C., Chen P.Y., Guo Y. (2013). Global climate change: Impact of diurnal temperature range on mortality in Guangzhou, China. Environ. Pollut..

[11] Vicedo-Cabrera A.M., Forsberg B., Tobias A., Zanobetti A., Schwartz J., Armstrong B., Gasparrini A. (2016). Associations of inter- and intraday temperature change with mortality. Am. J. Epidemiol..

[12] Ding Z., Guo P., Xie F., Chu H., Li K., Pu J., Pang S., Dong H., Liu Y., Pi F. (2015). Impact of diurnal temperature range on mortality in a high plateau area in southwest China: A time series analysis. Sci. Total Environ..

[13] Vutcovici M., Goldberg M.S., Valois M.F. (2014). Effects of diurnal variations in temperature on non-accidental mortality among the elderly population of Montreal, Quebec, 1984–2007. Int. J. Biometeorol..

[14] Lim Y.H., Reid C.E., Mann J.K., Jerrett M., Kim H. (2015). Diurnal temperature range and short-term mortality in large US communities. Int. J. Biometeorol..

[15] Song G., Chen G., Jiang L., Zhang Y., Zhao N., Chen B., Kan H. (2008). Diurnal temperature range as a novel risk factor for COPD death. Respirology.

[16] Kim J., Shin J., Lim Y.H., Honda Y., Hashizume M., Guo Y.L., Kan H., Yi S., Kim H. (2016). Comprehensive approach to understand the association between diurnal temperature range and mortality in east Asia. Sci. Total Environ..

[17] Cheng J., Xu Z., Zhu R., Wang X., Jin L., Song J., Su H. (2014). Impact of diurnal temperature range on human health: A systematic review. Int. J. Biometeorol..

[18] Gardner J.W., Sanborn J.S. (1990). Years of potential life lost (YPLL)—What does it measure?. Epidemiology.

[19] Huang C., Barnett A.G., Wang X., Tong S. (2012). The impact of temperature on years of life lost in Brisbane, Australia. Nat. Clim. Chang..

[20] Yang J., Ou C.Q., Guo Y., Li L., Guo C., Chen P.Y., Lin H.L., Liu Q.Y. (2015). The burden of ambient temperature on years of life lost in Guangzhou, China. Sci. Rep..

[21] Guo Y., Li S., Tian Z., Pan X., Zhang J., Williams G. (2013). The burden of air pollution on years of life lost in Beijing, China, 2004–08: Retrospective regression analysis of daily deaths. BMJ.

[22] He T., Yang Z., Liu T., Shen Y., Fu X., Qian X., Zhang Y., Wang Y., Xu Z., Zhu S. (2016). Ambient air pollution and years of life lost in Ningbo, China. Sci. Rep..

[23] Lu F., Zhou L., Xu Y., Zheng T., Guo Y., Wellenius G.A., Bassig B.A., Chen X., Wang H., Zheng X. (2015). Short-term effects of air pollution on daily mortality and years of life lost in Nanjing, China. Sci. Total Environ..

[24] Lim S.S., Vos T., Flaxman A.D., Danaei G., Shibuya K., Adair-Rohani H., Amann M., Anderson H.R., Andrews K.G., Aryee M. (2012). A comparative risk assessment of burden of disease and injury attributable to 67 risk factors and risk factor clusters in 21 regions, 1990–2010: A systematic analysis for the global burden of disease study 2010. Lancet.

[25] Naghavi M., Wang H., Lozano R., Davis A., Liang X., Zhou M., Vollset S.E.V., Abbasoglu Ozgoren A., Norman R.E., Vos T. (2015). Global, regional, and national age-sex specific all-cause and cause-specific mortality for 240 causes of death, 1990–2013: A systematic analysis for the global burden of disease study 2013. Lancet.

[26] Qian Z., He Q., Lin H.M., Kong L., Bentley C.M., Liu W., Zhou D. (2008). High temperatures enhanced acute mortality effects of ambient particle pollution in the “oven” city of Wuhan, China. Environ. Health Perspect..

[27] Zhang Y., Li C., Feng R., Zhu Y., Wu K., Tan X., Ma L. (2016). The short-term effect of ambient temperature on mortality in Wuhan, China: A time-series study using a distributed lag non-linear model. Int. J. Environ. Res. Public Health.

[28] Zhang Y., Yu C., Bao J., Li X. (2017). Impact of temperature variation on mortality: An observational study from 12 counties across Hubei province in China. Sci. Total Environ..

[29] Yang J., Ou C.Q., Song Y.F., Li L., Chen P.Y., Liu Q.Y. (2016). Estimating years of life lost from cardiovascular mortality related to air pollution in Guangzhou, China. Sci. Total Environ..

[30] Peng R.D., Dominici F., Louis T.A. (2006). Model choice in time series studies of air pollution and mortality. J. R. Stat. Soc. A.

[31] Kan H., London S.J., Chen H., Song G., Chen G., Jiang L., Zhao N., Zhang Y., Chen B. (2007). Diurnal temperature range and daily mortality in Shanghai, China. Environ. Res..

[32] Zhou X., Zhao A., Meng X., Chen R., Kuang X., Duan X., Kan H. (2014). Acute effects of diurnal temperature range on mortality in 8 Chinese cities. Sci. Total Environ..

[33] Gasparrini A., Armstrong B., Kenward M.G. (2010). Distributed lag non-linear models. Stat. Med..

[34] Gasparrini A. (2011). Distributed lag linear and non-linear models in R: The package dlnm. J. Stat. Softw..

[35] Guo Y., Gasparrini A., Armstrong B., Li S., Tawatsupa B., Tobias A., Lavigne E., de Sousa Zanotti Stagliorio Coelho M., Leone M., Pan X. (2014). Global variation in the effects of ambient temperature on mortality: A systematic evaluation. Epidemiology.

[36] Zhang Y., Yu C., Bao J., Li X. (2017). Impact of temperature on mortality in Hubei, China: A multi-county time series analysis. Sci. Rep..

[37] Zhao Q., Zhang Y., Zhang W., Li S., Chen G., Wu Y., Qiu C., Ying K., Tang H., Huang J.A. (2017). Ambient temperature and emergency department visits: Time-series analysis in 12 Chinese cities. Environ. Pollut..

[38] Lim Y.H., Park A.K., Kim H. (2012). Modifiers of diurnal temperature range and mortality association in six Korean cities. Int. J. Biometeorol..

[39] Zhou M., Wang H., Zhu J., Chen W., Wang L., Liu S., Li Y., Wang L., Liu Y., Yin P. (2016). Cause-specific mortality for 240 causes in China during 1990–2013: A systematic subnational analysis for the global burden of disease study 2013. Lancet.

[40] Cao J., Cheng Y., Zhao N., Song W., Jiang C., Chen R., Kan H. (2009). Diurnal temperature range is a risk factor for coronary heart disease death. J. Epidemiol..

[41] Magalhaes R., Silva M.C., Correia M., Bailey T. (2011). Are stroke occurrence and outcome related to weather parameters? Results from a population-based study in northern Portugal. Cerebrovasc. Dis..

[42] Benmarhnia T., Deguen S., Kaufman J.S., Smargiassi A. (2015). Review article: Vulnerability to heat-related mortality: A systematic review, meta-analysis, and meta-regression analysis. Epidemiology.

[43] Ban J., Xu D., He M.Z., Sun Q., Chen C., Wang W., Zhu P., Li T. (2017). The effect of high temperature on cause-specific mortality: A multi-county analysis in China. Environ. Int..

[44] Seposo X.T., Dang T.N., Honda Y. (2016). Effect modification in the temperature extremes by mortality subgroups among the tropical cities of the Philippines. Glob. Health Action.

[45] Onozuka D., Hagihara A. (2015). Variation in vulnerability to extreme-temperature-related mortality in Japan: A 40-year time-series analysis. Environ. Res..

[46] Basu R. (2009). High ambient temperature and mortality: A review of epidemiologic studies from 2001 to 2008. Environ. Health.

[47] Ding Z., Li L., Xin L., Pi F., Dong W., Wen Y., Au W.W., Zhang Q. (2016). High diurnal temperature range and mortality: Effect modification by individual characteristics and mortality causes in a case-only analysis. Sci. Total Environ..

[48] Lee M., Shi L., Zanobetti A., Schwartz J.D. (2016). Study on the association between ambient temperature and mortality using spatially resolved exposure data. Environ. Res..

[49] Zhang Y., Feng R., Wu R., Zhong P., Tan X., Wu K., Ma L. (2017). Global climate change: Impact of heat waves under different definitions on daily mortality in Wuhan, China. Glob. Health Res. Policy.

[50] Anderson B.G., Bell M.L. (2009). Weather-related mortality: How heat, cold, and heat waves affect mortality in the United States. Epidemiology.

