# Supplementary material for: Diurnal Temperature Range in Relation to Daily Mortality and Years of Life Lost in Wuhan, China
Source: Int J Environ Res Public Health. 2017 Aug 8;14(8):891. doi: 10.3390/ijerph14080891 (PMC5580595; doi:10.3390/ijerph14080891)
Supplement: Supplementary file 1 [file ijerph-14-00891-s001.pdf]

Supplementary Material

Table S1. Age-specific life expectancy for Chinese females and males for the year 2012.

Age Group (years)	Female	Male
<1	77.0	73.9
1-4	76.8	73.8
5-9	73.0	70.0
10-14	68.1	65.1
15-19	63.2	60.2
20-24	58.3	55.3
25-29	53.4	50.5
30-34	48.5	45.7
35-39	43.7	40.9
40-44	38.9	36.2
45-49	34.2	31.5
50-54	29.5	26.9
55-59	24.9	22.5
60-64	20.6	18.3
65-69	16.6	14.5
70-74	13.0	11.2
75-79	9.9	8.6
80-84	7.4	6.6
85-89	5.4	4.9
90-94	4.1	3.7
95-99	3.2	2.9
100+	2.8	2.3

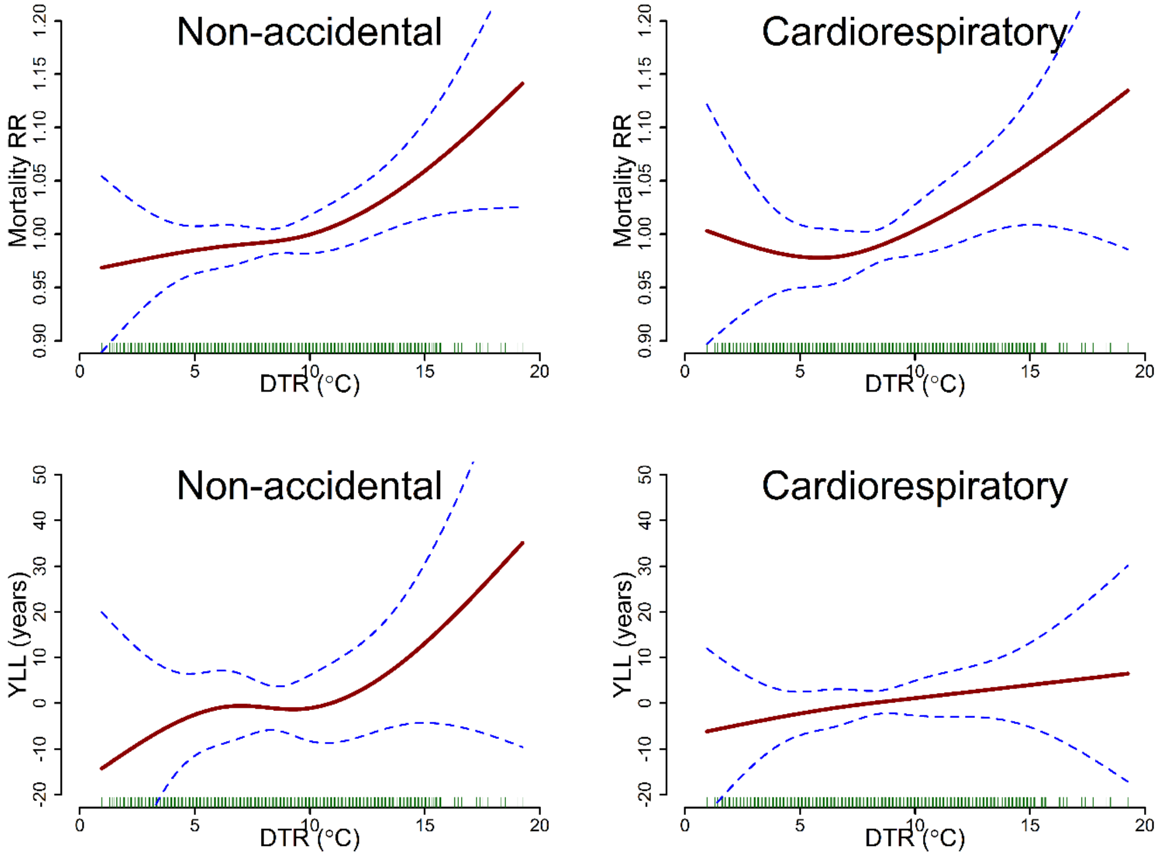

Figure S1. Dose-response relationships of DTR at lag 0–1 days (smoothing by a natural cubic spline with *df* = 3) with daily mortality and YLL due to non-accidental and cardiorespiratory deaths in Wuhan, China, 2009–2012. The continuous bold red lines represent the effect estimates and long-dashed blue lines are the 95% confidential intervals.

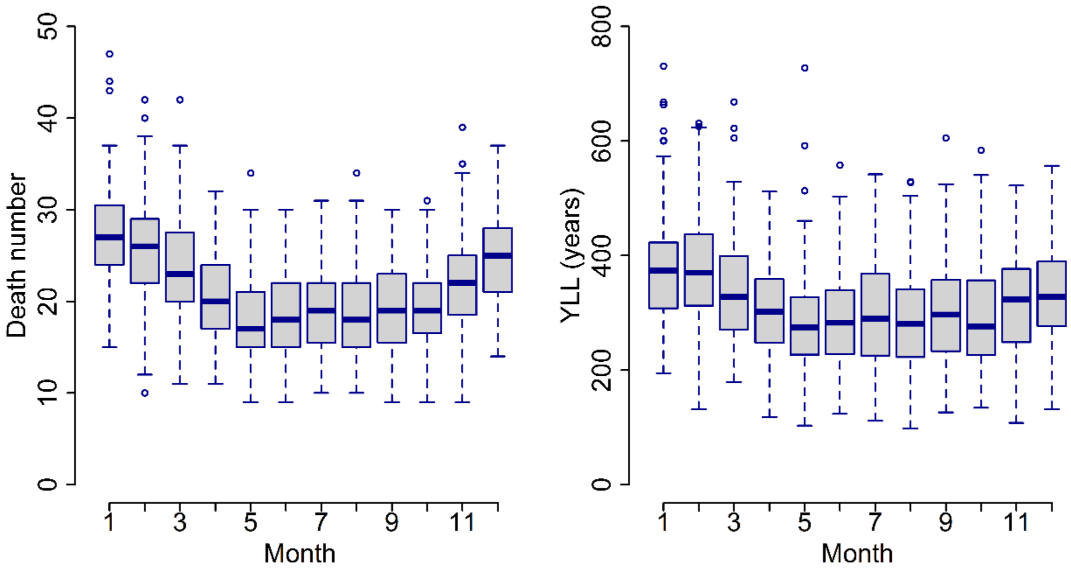

Figure S2. Boxplots for monthly death number and YLL due to non-accidental mortality in Wuhan, China, 2009–2012.

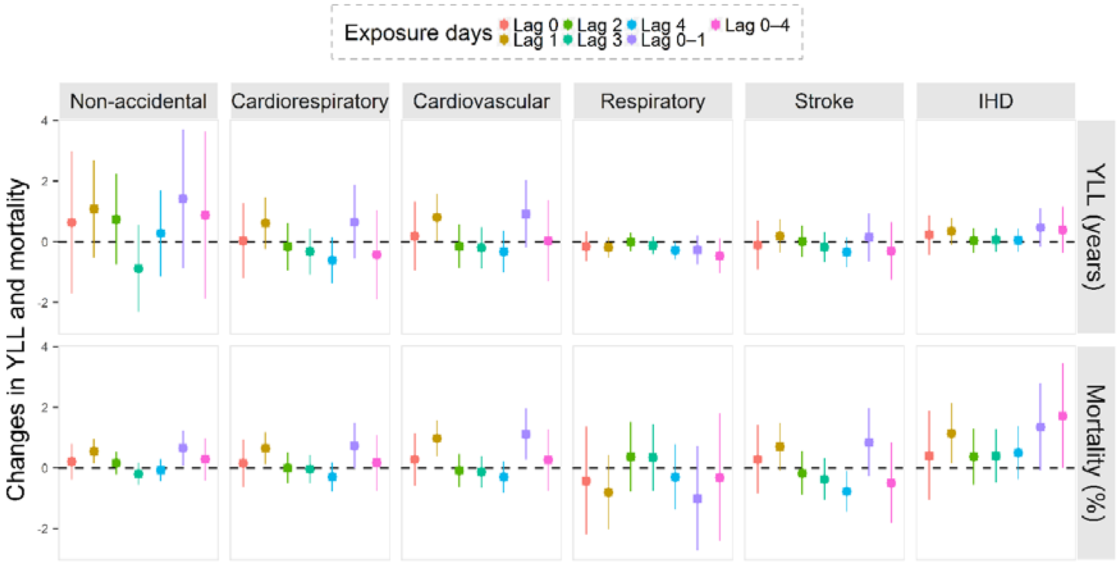

Figure S3. The estimated effects of DTR on cause-specific mortality and YLL at different lag days. The results were presented as changes in daily mortality (%) and YLL (years) associated with a 1 °C increase in DTR.

Table S2. Sensitivity analyses for DTR-associated effects on YLL and mortality among females, by changing *df* (4–6 per year) in the smoothness of calendar time and *df* (4–6) of natural cubic spline for mean humidity, mean wind speed, sunshine duration, and atmospheric pressure.

**Variables**	**Mortality (%)**		**YLL (years)**	
	**Non-accidental**	**Cardiovascular**	**Non-accidental**	**Cardiovascular**
Calendar time				
df = 4/year	1.26 (0.44, 2.09)	1.68 (0.54, 2.83)	1.51 (0.19, 2.84)	0.86 (0.22, 1.50)
df = 5/year	1.17 (0.34, 2.00)	1.64 (0.49, 2.79)	1.32 (−0.01, 2.66)	0.86 (0.21, 1.50)
df = 6/year	1.19 (0.35, 2.03)	1.70 (0.53, 2.87)	1.37 (0.02, 2.72)	0.88 (0.23, 1.54)
Mean humidity				
df = 4	1.24 (0.40, 2.09)	1.72 (0.54, 2.90)	1.44 (0.08, 2.80)	0.89 (0.23, 1.55)
df = 5	1.25 (0.40, 2.09)	1.73 (0.55, 2.91)	1.44 (0.08, 2.80)	0.90 (0.23, 1.56)
df = 6	1.25 (0.41, 2.10)	1.74 (0.56, 2.92)	1.46 (0.10, 2.82)	0.90 (0.24, 1.56)
Mean wind speed				
df = 4	1.30 (0.46, 2.15)	1.77 (0.59, 2.96)	1.57 (0.21, 2.93)	0.92 (0.26, 1.58)
df = 5	1.29 (0.44, 2.14)	1.85 (0.67, 3.04)	1.52 (0.16, 2.88)	0.95 (0.28, 1.61)
df = 6	1.30 (0.46, 2.15)	1.85 (0.67, 3.04)	1.54 (0.18, 2.90)	0.94 (0.28, 1.61)
Sunshine duration				
df = 4	1.26 (0.42, 2.11)	1.74 (0.57, 2.93)	1.42 (0.06, 2.78)	0.89 (0.23, 1.55)
df = 5	1.27 (0.43, 2.12)	1.72 (0.54, 2.90)	1.42 (0.06, 2.78)	0.87 (0.21, 1.53)
df = 6	1.28 (0.44, 2.13)	1.72 (0.55, 2.91)	1.44 (0.08, 2.80)	0.88 (0.22, 1.54)
Atmospheric pressure				
df = 4	1.27 (0.44, 2.12)	1.74 (0.56, 2.92)	1.48 (0.12, 2.84)	0.90 (0.24, 1.56)
df = 5	1.29 (0.45, 2.14)	1.77 (0.59, 2.96)	1.55 (0.19, 2.91)	0.93 (0.26, 1.59)
df = 6	1.30 (0.45, 2.14)	1.75 (0.57, 2.93)	1.56 (0.20, 2.92)	0.93 (0.27, 1.60)

Table S3. Sensitivity analyses for DTR-associated effects on YLL and mortality among the elderly (75+ years old), by changing *df* (4–6 per year) in the smoothness of calendar time and *df* (4–6) of natural cubic spline for mean humidity, mean wind speed, sunshine duration, and atmospheric pressure.

Variables	Mortality (%)		YLL (years)	
	Non-accidental	Cardiovascular	Non-accidental	Cardiovascular
Calendar time				
df = 4/year	0.72 (0.00, 1.44)	1.25 (0.26, 2.25)	0.72 (0.07, 1.37)	0.63 (0.18, 1.09)
df = 5/year	0.64 (−0.09, 1.37)	1.19 (0.19, 2.20)	0.65 (0.00, 1.31)	0.61 (0.15, 1.06)
df = 6/year	0.68 (−0.06, 1.42)	1.20 (0.19, 2.23)	0.70 (0.04, 1.36)	0.60 (0.14, 1.07)
Mean humidity				
df = 4	0.70 (−0.04, 1.44)	1.20 (0.18, 2.23)	0.71 (0.04, 1.37)	0.59 (0.12, 1.06)
df = 5	0.70 (−0.03, 1.45)	1.21 (0.19, 2.24)	0.72 (0.05, 1.39)	0.60 (0.13, 1.06)
df = 6	0.70 (−0.04, 1.45)	1.22 (0.20, 2.25)	0.72 (0.05, 1.39)	0.60 (0.13, 1.07)
Mean wind speed				
df = 4	0.73 (−0.01, 1.48)	1.24 (0.21, 2.27)	0.73 (0.06, 1.40)	0.60 (0.14, 1.07)
df = 5	0.76 (0.02, 1.51)	1.30 (0.27, 2.34)	0.76 (0.09, 1.43)	0.63 (0.16, 1.10)
df = 6	0.77 (0.03, 1.52)	1.30 (0.28, 2.34)	0.77 (0.10, 1.44)	0.63 (0.17, 1.10)
Sunshine duration				
df = 4	0.71 (−0.03, 1.46)	1.23 (0.21, 2.26)	0.72 (0.05, 1.38)	0.60 (0.14, 1.07)
df = 5	0.72 (−0.02, 1.47)	1.20 (0.18, 2.23)	0.73 (0.06, 1.40)	0.59 (0.12, 1.06)
df = 6	0.72 (−0.02, 1.47)	1.20 (0.18, 2.23)	0.73 (0.06, 1.40)	0.59 (0.12, 1.06)
Atmospheric pressure				
df = 4	0.73 (0.00, 1.48)	1.23 (0.21, 2.26)	0.74 (0.08, 1.41)	0.61 (0.14, 1.07)
df = 5	0.73 (−0.01, 1.47)	1.22 (0.20, 2.25)	0.74 (0.07, 1.41)	0.61 (0.14, 1.07)
df = 6	0.73 (−0.01, 1.48)	1.21 (0.19, 2.25)	0.74 (0.07, 1.41)	0.60 (0.13, 1.07)

Table S4. Yearly summary distributions of daily death number and YLL during 2009–2012.

Year	Daily Deaths			Daily YLL (years)		
	Mean	SD	Range	Mean	SD	Range
2009	21.3	5.9	9–44	330.1	95.6	97.9–730.8
2010	21.3	5.9	10–43	320.0	97.1	132.2–668.4
2011	21.5	6.0	9–47	315.6	103.0	103.1–727.6
2012	21.5	5.8	9–39	305.4	91.7	126.1–667.8
2009–2012	21.4	5.9	9–47	317.8	97.2	97.9–730.8
